# Sex/Gender Differences in Metabolism and Behavior: Influence of Sex Chromosomes and Hormones

**DOI:** 10.1155/2015/245949

**Published:** 2015-09-28

**Authors:** Haifei Shi, Lynda M. Brown, Roshanak Rahimian

**Affiliations:** ^1^Department of Biology, Miami University, Oxford, OH 45056, USA; ^2^Foods and Nutrition Science Program, North Carolina A&T State University, Greensboro, NC 27411, USA; ^3^Department of Physiology and Pharmacology, Thomas J. Long School of Pharmacy & Health Sciences, University of the Pacific, Stockton, CA 95211, USA

Mammalians, including mice, rats, pigs, and dogs, used in scientific research are often males. Such bias is evident because researchers avoid using female animals as they are concerned that their reproductive cycles and hormone fluctuations may confound the results of their studies, which may lead to some disparity observed between preclinical studies and clinical trials. The National Institutes of Health (NIH) in the United States has been directing basic and clinical scientists to consider potential sex differences and perform their studies using cells and tissues derived from both males and females and using both male and female subjects since 2014. We expect an increase in research attention on study of sex differences during the upcoming era.

The research scope for studying the influence of sex hormones has been traditionally focused on the regulation of physiological and behavioral processes as well as dysregulation and subsequent disorders related to the reproductive system. However, sex/gender differences are not limited to their impact on the reproductive system. Sex chromosomes and hormones affect the structure, physiology, and function of various organ systems throughout the lifespan, with impact of hormonal changes at different life stages, such as puberty, pregnancy, aging, and menopause. Indeed, significant sex-based differences exist in the regulation of diverse behavior and metabolism as well as the development and progression of varied diseases, such as obesity, diabetes, and some types of renal, hepatic, and psychiatric disorders, involving multiple systems. For example, women have lower risks for obesity-related metabolic disorders than men; however, the prevalence of these metabolic disorders increases dramatically in women after menopause. Furthermore, cardiovascular diseases are less common in premenopausal women compared with age-matched men, but this difference disappears in the postmenopausal years.

Less attention has been paid to the sex-based differences in the systems other than reproductive system and to the potential influence of sex hormones in the above mentioned diseases. Despite major advances in our understanding of the effects of sex chromosomes and hormones on physiological processes and functions and the potential roles of sex chromosomes and hormones in the development and progression of diseases, many knowledge gaps and paradoxes remain. A better understanding of the mechanisms underlying the sex-based differences in behavior/metabolism and of the influences of sex chromosomes and hormones is expected to lead to novel therapeutic strategies that would benefit patients of both sexes and enhance the concept of individualized medicine. Thus, adequate representation of both sexes in the basic and translational preclinical studies should be provided, as emphasized by the NIH recent requirement intended to minimize sex biases in preclinical research.

The goal of this special issue is to stimulate the continuing efforts to demonstrate the breadth of the behavioral, physiological, cellular, and molecular processes beyond the reproductive system which exhibit sex-based differences under physiological and pathological conditions, with a particular concentration on sex chromosomes and hormones. This special issue showcases insightful reviews of up-to-date literature as well as innovative studies that illustrate important contribution of sex hormones to the physiological and behavioral processes both centrally and peripherally in metabolism and behavior which are not limited to the reproductive system.

In the central nervous system, sex hormones regulate energy homeostasis (X. Liu and H. Shi) and neuropsychiatric process (A. Gogos et al.), with malfunction contributing to metabolic disorders, schizophrenia, and other psychiatric disorders such as depression and anxiety. In periphery, sex hormones, especially estrogens, have potential protective effects on glucose and lipid metabolism in the liver (M. Shen and H. Shi) and on perfusion pressure of the kidney (A. Zúñiga-Muñoz et al.), with dysregulation leading to hepatic and renal dysfunction. Additionally, sex hormones play critical roles in the development and progression of many types of cancer, such as hepatocellular carcinoma associated with hepatitis B virus infection (M. Montella et al.) and lung adenocarcinoma (D. Stakisaitis et al.).

We are pleased to serve as Guest Editors of this special issue. Despite the sex-associated differences in physiological processes and functions as well as pathological development and progression of diseases, research has predominantly involved male subjects, and many knowledge gaps and paradoxes still remain. We hope that this special issue will help raise awareness of sex-based differences in multiple systems under healthy conditions and diseases conditions, guide researchers for their experimental designs using both male and female subjects and samples, and advance the understanding of underlying mechanisms of sex differences in behavior and metabolism involving multiple systems.

*Haifei Shi* *Haifei Shi* *Lynda M. Brown* *Lynda M. Brown* *Roshanak Rahimian* *Roshanak Rahimian*

